# Atypical Manifestation of Acute Myocardial Infarction (AMI) in a Vicenarian Woman With Polycystic Ovary Syndrome (PCOS) Undergoing Assisted Reproductive Technology (ART) Treatment: A Case Report

**DOI:** 10.7759/cureus.57537

**Published:** 2024-04-03

**Authors:** Nikita Hulke, Shilpa Dutta, Avinash B Taksande

**Affiliations:** 1 Physiology, School of Allied Health Sciences, Datta Meghe Institute of Higher Education & Research, Wardha, IND; 2 Clinical Embryology, School of Allied Health Sciences, Datta Meghe Institute of Higher Education & Research, Wardha, IND; 3 Physiology, Jawaharlal Nehru Medical College, Datta Meghe Institute of Higher Education & Research, Wardha, IND

**Keywords:** neuro physiotherapy, ovarian cyst, in vitro fertilization [ivf], acute myocardial infarcation, polycystic ovary syndrome (pcos)

## Abstract

Polycystic ovary syndrome (PCOS) is a prevalent endocrinological disorder affecting women of reproductive age, characterized by hormonal imbalances leading to metabolic and reproductive dysregulations. Acute myocardial infarction (AMI) represents a critical cardiovascular event, traditionally observed in older populations but increasingly identified in younger individuals with diverse medical backgrounds. The pursuit of assisted reproductive technology (ART) by women with PCOS to address infertility may further complicate cardiovascular risks due to the exogenous hormonal manipulations involved. This case report delineates a rare presentation of AMI in a 27-year-old vicenarian woman with PCOS undergoing ART treatment. Despite the absence of conventional cardiovascular risk factors, the patient exhibited typical symptoms and diagnostic features of AMI. Prompt recognition and intervention facilitated successful management and favorable outcomes. This case underscores the importance of considering atypical cardiovascular presentations in young women with complex medical histories, necessitating heightened awareness among healthcare providers. Multidisciplinary collaboration is imperative for comprehensive risk assessment, prevention, and tailored management strategies in this population. Further research is warranted to elucidate the intricate interplay between PCOS, ART, and cardiovascular outcomes, thereby optimizing clinical care and enhancing reproductive outcomes in this vulnerable cohort. An enhanced understanding of these relationships is essential for guiding evidence-based interventions aimed at mitigating cardiovascular risks and improving overall health outcomes in women with PCOS undergoing fertility treatments.

## Introduction

The most common etiologically challenging pathology that has disrupted the lives of females in the reproductive age group is polycystic ovary syndrome (PCOS). It has been found that around 5 to 15% of women suffer from PCOS worldwide currently [1]. Out of which, 3.7 to 22.5% of the population prevalence is present in India [2]. It has various significant clinical complications associated with it, like metabolic aberrations such as insulin resistance, dyslipidemia, hypertensive disorders, psychological problems like anxiety, disruptive quality of life, and reproductive disability, which are the major manifestations of this syndrome [3,4]. Although PCOS is most commonly recognized for its detrimental impact on conception, it is also known to cause an assortment of metabolic disorders that put affected people at risk for cardiovascular illnesses. Furthermore, there has been increasing research suggesting that PCOS may be correlated with an elevated likelihood of obesity, dyslipidemia, insulin resistance, and endothelial dysfunction, a condition closely related to cardiovascular disease (CVD) [5]. Acute myocardial infarction (AMI), typically defined as a heart attack in layman’s language, is often manifested by a decrement or cessation of blood supply in a portion of the heart, which may lead to the death of the cells of the heart muscles. This is generally caused by the presence of blood clots in the epicardial artery, which is typically known to supply the cardiac muscles [6]. Traditionally, it has been seen that AMI has risk factors like smoking, obesity, advanced age, and dyslipidemia [7]. Nonetheless, a steadily increasing amount of study evidence in scientific journals emphasizes that AMI may manifest atypically, in particular among younger people with varied medical backgrounds and comorbidities.

Women with PCOS often resort to in vitro fertilization treatment to get assistance in conception, as it has been seen that PCOS females often face anovulatory syndrome [8]. Hence, this population may be more predisposed to CVD due to the anxiety driven by infertility interventions, hormonal alterations, and the underpinning metabolic abnormalities of PCOS. In spite of this, there nevertheless exists a dearth of evidence in the literature addressing the hyperlink between PCOS, assisted reproductive technology (ART), and AMI, and healthcare professionals are not fully apprised of the prospect of atypical AMI presentations in these circumstances. The present case report depicts a bizarre AMI presentation in a vicenarian woman undergoing ART who had a history of PCOS. The relevance of implementing consideration of alternative risk factors and clinical presentations in younger patients, especially those with convoluted medical histories and ongoing medical interventions, is stressed in this article. By elucidating the subtleties of this particular situation, we aim to raise the level of knowledge among medical professionals regarding the various ways that AMI might present in distinct patient groups, which will eventually enable early detection and better treatment approaches.

## Case presentation

A 27-year-old nulliparous patient presented at the emergency department of a tertiary healthcare center situated in Sawangi, Maharashtra, with chief complaints of a severe sudden onset of epigastric pain radiating toward her back torso along with profuse diaphoresis and nausea. The patient had no significant manifestation of any diseases in the past, except for the diagnosis of PCOS in the past six years. The patient had been married for three years and was typically undertaking infertility treatment at the Wardha Test Tube Baby Centre located in Wardha, Maharashtra, which includes ovarian stimulation using synthetic gonadotrophins as a prototype of the IVF treatment. The patient had no history of smoking or alcoholism and had no prior history of hypertension, diabetes mellitus, etc. Initial assessment of the patient upon presentation to the department revealed distress and diaphoresis. Vital signs revealed a body temperature of 37.3 °C (normal temperature: 37 °C), blood pressure of 145/92 mmHg (normal blood pressure: 120/80 mmHg), heart rate of 113 beats/minute (normal heart rate: 60 to 100 beats per minute), respiratory rate of 21 breaths/minute (normal respiratory rate: 12 to 16 breaths per minute), and blood oxygen saturation (SpO_2_) of 98% in room air (normal range: >95%). Cardiac auscultation revealed a regular rhythm with no murmurs or added sounds. The body mass index of the patient was 28.5 kg/m^2^ (normal range: 18.5 to 24.9 kg/m^2^). The analysis of urine drug screening was negative. A contrast-enhanced CT scan of the thoracic spine revealed no evidence of an epidural abscess, fracture, or misalignment. CT angiography of the chest, abdomen, and pelvis also revealed no evidence of pulmonary embolism, aortic aneurysm, or dissection. Laboratory investigations demonstrated elevated cardiac biomarkers, with troponin I levels peaking at 15.4 ng/mL (the normal range is less than 0.04 ng/mL). Table 1 includes the blood investigation report for the patient.

**Table 1:** Blood investigation report of the patient HDL, high-density lipoprotein; LDL, low-density lipoprotein; TSH, thyroid-stimulating hormone

Parameters	Patient report	Reference range
Platelets (per mm^3^)	311,200	182,000-369,000
White cell count (per mm^3^)	18,544	4,000-11,000
Neutrophils (%)	81.8	34.0-71.1
Lymphocytes (%)	14.1	19.3-51.7
Monocytes (%)	9.3	4.7-12.5
Eosinophils (%)	0.1	0.7-5.8
Hemoglobin (g/dL)	12.9	11.2-15.7
Troponin I (ng/mL)	15.4	0.000-0.034
Magnesium (mg/dL)	1.8	1.6-2.3
Lactic acid (mmol/L)	1.4	0.7-2.1
Random blood sugar (mg/dL)	112	<140
Creatinine (mg/dL)	0.8	0.7-1.2
Potassium (mmol/L)	4.3	3.5-5.1
Sodium (mmol/L)	139	134-145
TSH (mIU/L)	2.12	0.47-4.68
C-reactive protein (mg/L)	0.4	<3
Cholesterol (mg/dL)	234	<200
Triglycerides (mg/dL)	139	<150
HDL (mg/dL)	38	40-59
LDL (mg/dL)	179	100-129

**Figure 1:**
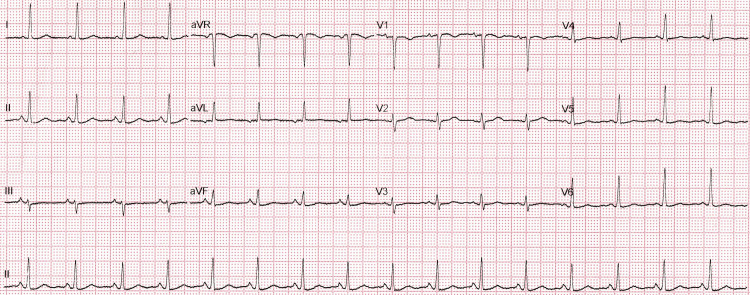
ECG revealed ST-segment elevation in leads II, III, and aVF ST-segment: the period between the end of ventricular depolarization and the beginning of ventricular repolarization on the ECG aVF, augmented vector foot

ECG revealed ST-segment elevation in leads II, III, and aVF, consistent with an acute inferior wall myocardial infarction (AMI). Figure 1 depicts the report of the ECG.

Patient medical history

The patient was diagnosed with a striking medical history of PCOS, which was determined through ultrasound results that indicated bilateral polycystic ovaries, anovulation, and clinical signs of hyperandrogenism. Her menstrual cycles had been well controlled with lifestyle changes and oral contraceptive tablets. However, due to being unable to procure conception through natural intercourse for the last two out of the three years of their married life, they sought infertility treatment six months ago and underwent one cycle of ovarian stimulation. However, the result was negative. Also, the patient did not have any family history of hypercoagulable syndromes or any hereditary disorders. The hypercoagulable workup was also reported to be within the normal range.

Treatment

The patient was promptly started on dual antiplatelet medication with aspirin and a P_2_Y_12_ inhibitor (clopidogrel) upon diagnosis of AMI. Given her unusual appearance and the possibility of underlying PCOS-related endothelial dysfunction, she was also started on atorvastatin, a high-intensity statin, for lipid-lowering therapy. After the patient had emergency coronary angiography due to a substantial ST-segment elevation on the ECG, the procedure identified a right coronary artery proximal blockage. The successful implantation of a stent during percutaneous coronary intervention (PCI) restored coronary blood flow. The patient was moved to the cardiac intensive care unit for close observation and further care after PCI. The patient underwent extensive cardiac rehabilitation counseling in addition to medication and interventional therapy. The counseling focused on lifestyle improvements, such as dietary adjustments, frequent exercise, and stress management methods. Additionally, preconception counseling was started in coordination with experts in reproductive endocrinology and obstetrics.

Follow-up

Following the procedure, the patient’s ailments abated, and her hemodynamic stability persisted. Troponin levels diminished with time, which was compatible with the resolution of myocardial infarction, according to serial cardiac enzyme assays. The patient was released with a prescription (aspirin 85 mg daily, atorvastatin 75 mg daily, metoprolol 60 mg twice a day, nitroglycerine 0.4 mg sublingual as needed, and prasugrel 10 mg daily), lifestyle changes, and cardiac rehabilitation. The patient had a satisfactory recovery, and there was no indication of any negative cardiovascular outcomes or repeated ischemia episodes during follow-up visits at the cardiology clinic.

## Discussion

The case report sheds light on the complex interaction among cardiovascular health, ART, and PCOS. This is especially relevant in light of the unusual presentation of AMI in a vicenarian woman undergoing ART treatment. With a frequency ranging from 4% to 12% worldwide, PCOS has been regarded as one of the most prevalent endocrinological issues affecting women in the reproductive age group. There are four main diagnostic criteria currently used for the diagnosis of PCOS: ovulatory dysfunction, hirsutism, hyperandrogenism, and numerous ovarian cysts on ultrasound [9].

It is the most prevalent root cause of metabolic inconsistencies, hirsutism, anovulatory infertility, and obesity. Furthermore, it elevates the long-term risk of endometrial cancer, type 2 diabetes mellitus, obesity, anxiety, and potentially CVD [9]. In advanced nations, AMI is one of the most prevalent causes of mortality. Over a million human beings perish from the condition annually in the United States, where its prevalence is roughly three million [10]. It has been reported that Indians are four times more likely to develop AMI in their lifetime than those from other nations because of a confluence of lifestyle and genetic variables that encourage metabolic disorders [11]. There are instances of 31.7% of deaths in India currently due to AMI [11]. Several studies have shown that AMI is generally associated with higher age groups [9,12].

However, the latest studies show an association between AMI and younger age groups as well [9,13]. Several studies have reported controversial findings regarding the association between COS and CVD. However, a meta-analysis of 10 studies involving 104,392 subjects revealed a significant link between PCOS and increased CVD risk (OR = 1.30; 95% CI: 1.09-1.56; P = 0.004), particularly with coronary heart disease (OR = 1.44; 95% CI: 1.13-1.84; P = 0.004) [14]. As PCOS is known to trigger chronic inflammation, oxidative stress, impaired fibrinolysis, endothelial dysfunction, increased intimal thickness, increased coronary artery calcification, and diminished pulse wave velocity, it boosts the risk of coronary artery disease [9]. Also, it has been reported that oral contraceptives typically affect practically all homeostatic parameters, such as the plasma level of coagulation factors, anticoagulant proteins, and proteins implicated in fibrinolytic pathways; they also further elevate the risk of thromboembolic events and AMI [15]. This case underscores the importance of vigilance among medical practitioners regarding atypical symptoms and risk factors for CVD in reproductive-aged women, especially those undergoing fertility treatments. The coexistence of PCOS, characterized by hormonal dysregulation and metabolic disturbances, and ART procedures, which impose additional physiological stressors, may synergistically increase the risk of cardiovascular events such as AMI. Therefore, a comprehensive approach to cardiovascular risk assessment and management is imperative for this population. Future research endeavors should focus on elucidating the underlying mechanisms linking PCOS, ART, and CVD to inform evidence-based guidelines and improve clinical outcomes in this vulnerable population.

## Conclusions

This case highlights the necessity of recognizing nontraditional presentations of AMI in young women with intricate medical histories, particularly those with PCOS undergoing ART treatment. It emphasizes the critical role of healthcare providers in maintaining heightened awareness for the timely identification and management of cardiovascular events in this distinctive patient demographic. Collaboration among cardiologists, endocrinologists, and reproductive specialists is imperative to optimize risk assessment, implement preventive measures, and tailor therapeutic interventions to the specific needs of PCOS-afflicted women undergoing fertility treatments. However, given the singular nature of this case, further investigations are necessary to elucidate the underlying mechanisms linking PCOS, ART, and cardiovascular outcomes. Such endeavors aim to enhance our understanding of these complex interactions, ultimately leading to improved cardiovascular health and reproductive outcomes in this vulnerable population. Future research efforts should focus on elucidating the pathophysiological pathways linking PCOS, ART, and cardiovascular events, thereby informing evidence-based interventions and enhancing clinical care for women undergoing fertility treatments.
